# Targeted Prostate Health Checks, a novel system to identify men with prostate cancer—A pilot study

**DOI:** 10.1002/bco2.416

**Published:** 2024-08-15

**Authors:** Stephen Langley, Jeremy Goad, Stephanie Bell, Lee Foster, Catherine Hodges, Marc Laniado, Michele Pietrasik, Alison Rooke, Matthew Knight, Edward Bosonnet, Simon Bott

**Affiliations:** ^1^ The Stokes Centre for Urology Royal Surrey Hospital NHS Foundation Trust Guildford UK; ^2^ Medefer Ltd London UK; ^3^ Surrey and Sussex Cancer Alliance Guildford UK; ^4^ Surrey Primary Care Lead Surrey & Sussex Cancer Alliance Guildford UK; ^5^ Wexham Park Hospital Slough Berkshire UK; ^6^ Frimley Park Hospital, Frimley Health NHS Foundation Trust Frimley UK

**Keywords:** health check, multiparametric‐MRI, prostate cancer, prostate‐specific antigen, PSA, transperineal biopsy

## Abstract

**Objectives:**

The objective of this study is to report the pilot phase of the Targeted Prostate Health Check programme that aims to identify men in the Surrey and Sussex region who have prostate cancer and who failed to be detected during the Covid era.

**Subjects and methods:**

Men aged 50 to 70, or 45 to 70 if Black or with a family history of prostate cancer, were identified from participating general practitioner (GP) records. Short message service (SMS) texts invited men to visit www.talkprostate.co.uk for information on prostate cancer and give consent to prostate‐specific antigen (PSA) checks coordinated by a third‐party virtual healthcare provider. Elevated age‐related PSA levels, or levels below age‐related thresholds but at 3 ng/mL or more, triggered referral to a rapid access urology clinic. GPs were informed of the results.

**Results:**

From 1842 text messages inviting 1549 people, 544 men consented to a PSA check. From 500 phlebotomy appointments, 485 (30% of invited men) took the PSA test of whom 68 (14%) were referred with an elevated PSA. After clinical review with multiparametric magnetic resonance imaging (mp‐MRI), 22 patients underwent transperineal biopsies, and prostate cancer was detected in 18 men of whom 17 (95%) had clinically significant cancer.

**Conclusion:**

Our Targeted Prostate Cancer Health Check system identifies men at risk without burdening primary care. Awareness on prostate cancer risk was raised in 1549 invited men, half of whom were further educated via the registration website. One third of invited men were checked in whom clinically significant prostate cancer was found in 3.5%.

## 1 INTRODUCTION

The Surrey and Sussex Cancer Alliance (SSCA)^1^ covers a population of 3.2 million people in the South of England. Its mission is to shape, deliver and oversee a programme of interventions to improve cancer detection, diagnosis, treatment outcomes and patient experience in line with National Health Service (NHS) guidance.

During the Covid pandemic, the number of men who presented to primary care for routine health consultation relating to prostate concerns fell, as did the number of men subsequently referred with suspected prostate cancer. Between March 2020 and December 2022, the SSCA saw 1334 fewer referrals of men suspected of prostate cancer and 656 fewer diagnoses. To address the effect of the Covid pandemic on cancer detection and improve access to prostate‐specific antigen (PSA) testing, the SSCA created the Targeted Prostate Health Check programme. With funding from NHS England (NHSE) and developed together with NHS partners and Medefer, an NHS‐accredited independent sector virtual healthcare provider,^2^ the programme aims to identify men at risk of prostate cancer with at least 10 years of life expectancy and offer PSA testing without burdening primary care whilst keeping GPs informed regarding outcomes. The case‐finding methodology uses GP records to search for males with risk factors based on age, ethnicity, and family history, to detect early yet significant cancer and thereby reduce prostate cancer‐related mortality.

Guidance in the United Kingdom (UK) on age thresholds for testing and the PSA values that trigger referral for prostate cancer at the primary care level has been confusing. The National Institute for Health and Care Excellence (NICE) has endorsed age‐specific thresholds ranging from 2.5 to 6.5 ng/mL in symptomatic men,^3^ whereas NHSE^3^ has recommended asymptomatic men with a PSA ≥ 3 ng/mL be referred for urgent assessment in line with European Association of Urology (EAU) recommendations.^4^ Furthermore, in England, asymptomatic men aged 50 and over can approach their GPs to discuss PSA testing. However, advice from the NHS Office for Health Improvement and Disparities stated that GPs should not proactively raise the issue with asymptomatic men,^5^ yet early prostate cancer is usually symptomless.^6^ GPs have differing opinions regarding the value of PSA levels in asymptomatic men.^7^ This has led to men themselves seeking PSA testing, often in unregulated services such as sports clubs or other social venues.

The main objection to PSA testing in asymptomatic men or in the general population has related to overdiagnosis and overtreatment of otherwise indolent disease. However, the prostate cancer diagnostic pathway has been transformed over the last few years by integration of magnetic resonance imaging (MRI). The use of multiparametric MRI (mp‐MRI) has been shown to reduce the need for biopsy of men with raised PSA, reduce detection of clinically insignificant cancer and identify over 90% of clinically significant cancers.^8^ Practice‐changing level I evidence from the European Randomized Study of Screening for Prostate Cancer (ERSPC)^9^ and the Göteborg Randomized Population‐Based Prostate Cancer Screening Trial^10^ did not use pre‐biopsy MRI and relied upon the transrectal biopsy technique for diagnosis but still demonstrated the value of PSA testing in reducing prostate cancer mortality. Targeted transperineal biopsy techniques performed under local anaesthetic have shown to be more accurate in detecting clinically significant cancer with significantly reduced infectious complications compared with a transrectal biopsy.^11^, ^12^ The above screening studies were also undertaken before the widespread adoption of active surveillance that allows radical treatment to be postponed, perhaps indefinitely, whilst undertaking regular monitoring with PSA, mp‐MRI and infrequent biopsies. This modern management strategy has done much to reduce overdiagnosis, unwarranted radical treatment and attendant morbidity for men with low‐risk localised cancer. The SSCA has adopted this strategy, and consequently only men with a suspicious mp‐MRI proceed to transperineal prostate biopsy.

The Targeted Prostate Health Check programme grant from NHSE supports PSA tests on up to 22 000 men over a 2‐year period. The pilot commenced 15th August 2022 and reports on circa 500 men attending PSA checks at designated GP practices in the Guildford area with referrals to the Royal Surrey Hospital. Data from the pilot study inform the roll out of the project across the Alliance and bring us closer to finding the ‘missing’ men unaccounted for during the Covid pandemic from March 2020 to December 2022.

## 2 SUBJECTS AND METHODS

### 2.1 Case identification

**FIGURE 1:**
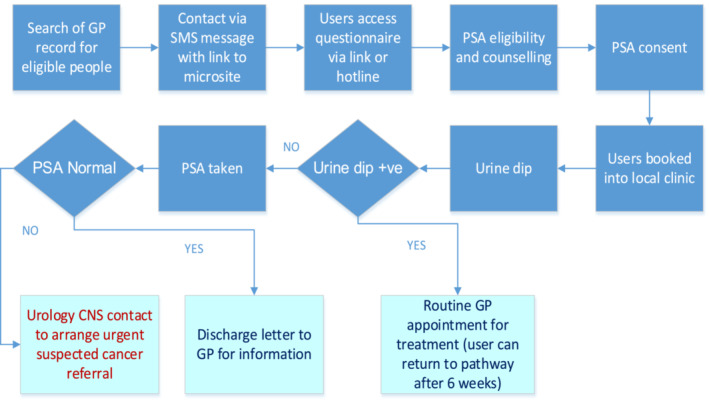
Pathway for Targeted Prostate Health Checks. CNS, clinical nurse specialist; GP, general practitioner; PSA, prostate‐specific antigen; SMS, short message service.

Participating GP databases identified eligible men aged 50 to 70 or 45 to 70 if black or with a family history of prostate cancer. They were contacted via short message service (SMS) texts and directed to a dedicated website (www.talkprostate.co.uk). The website provides information on prostate cancer and the potential risks and benefits of PSA testing as recommended by the Public Health England Prostate Cancer Risk Management Programme.^6^ It also allows service feedback by means of a survey as well as assessing patients' understanding of prostate cancer. Patients are able to register through the website for a PSA test and give consent. For those without access to the internet, the service can also be accessed by telephone. Eligible patients are booked to have a PSA test at an out of hours community clinic coordinated by Medefer staff. In the near future, a mobile clinic will also become available. Patients are advised to avoid strenuous exercise and sexual activity for 3 days before their PSA test. On arrival at the clinic, a urine sample is assessed by dipstick for infection and if clear a blood sample is drawn. Where a urinary infection is detected, patients are directed to see their GP and a subsequent test date arranged 6–8 weeks hence. Patients and GPs are informed by email if the PSA was considered normal. If PSA results are not within the specified ranges, patients are telephoned by a Medefer urology clinical nurse specialist (CNS) and undergo direct referral into the local Urology Department Rapid Access Clinics on the Urgent Suspected Cancer (USC) referral pathway. For the purpose of the pilot study, this was at the Royal Surrey Hospital, Guildford, where clinical review, mp‐MRI and transperineal biopsies were performed following the local prostate cancer detection protocol. The steps of the Targeted Prostate Health Check pathway at GP level can be seen in Figure 1.

### 2.2 Prostate cancer management protocol

Patients referred with an elevated PSA were assessed by initial mp‐MRI followed by clinical review and examination. Cognitive targeted transperineal prostate biopsy with systematic sampling (10–12 cores) under local anaesthetic using the Precision Point technique was undertaken when biopsy was indicated, as described in the Faster Diagnostic Pathway for Prostate Cancer, when the mp‐MRI was Prostate Imaging–Reporting and Data System (PI‐RADS) ≥3.^13^ Clinically significant cancer was defined by a Gleason ≥7 (WHO/International Society of Urological Pathology Gleason Grade Group [GG] ≥ 2).^14^, ^15^

## 3 RESULTS

**FIGURE 2:**
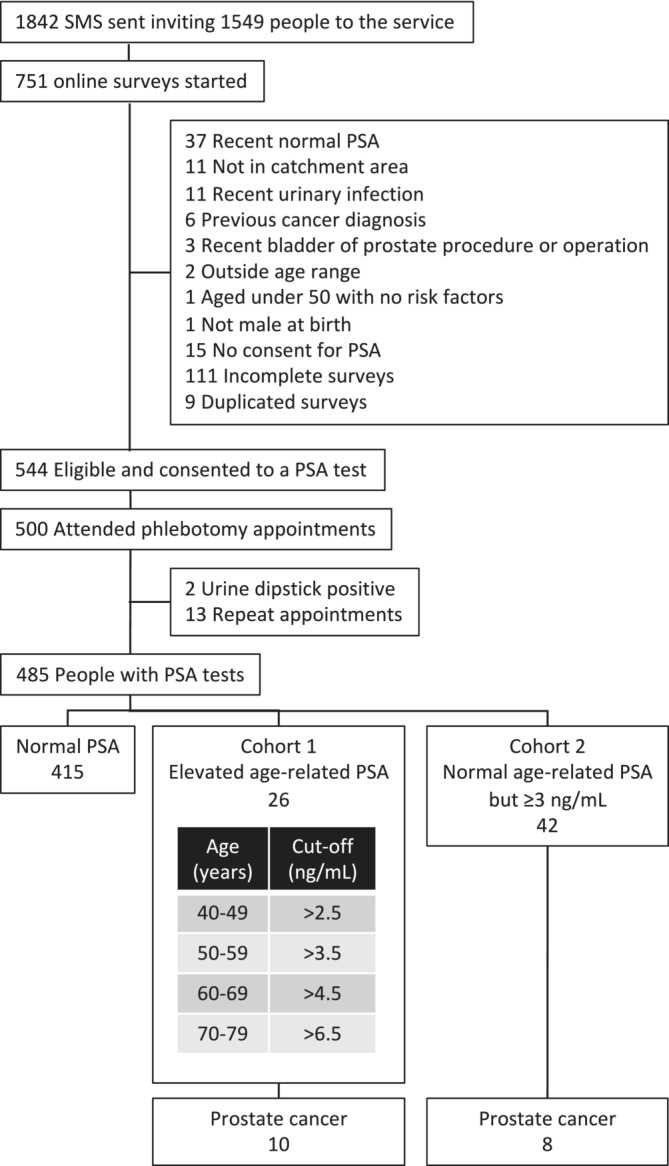
Flow chart of participants from receiving initial short message service (SMS) text to prostate cancer diagnosis. PSA, prostate‐specific antigen.

For this pilot study, 1549 men identified from participating GP practices received SMS texts inviting them to the service and to view the website (Figure 2). Those identified as being at higher risk were sent reminder texts (1842 texts in all). Of these, 751 registered on the website and started to fill out the survey but not all men qualified for the service, for example, 4.9% had a recent normal PSA, 1.5% were not in the pilot catchment area, 1.5% had a recent urinary infection, 0.8% had a previous (non‐prostate) cancer diagnosis, 0.4% had a recent bladder or prostate procedure or operation, 0.3% were outside the age range, 0.1% aged under 50 with no risk factors and 0.1% were not male at birth. Fifteen (2%) cases declined a PSA test after reviewing the information on the website. In all, 544 men met the criteria for inclusion in the programme and consented to a PSA test. Of these, a family history was documented in 17.9% participants, 2.3% black ethnicity and both black ethnicity plus family history in 0.4%. The telephone hotline was used by 1.3% of participants in order to complete the survey. From 500 phlebotomy appointments, two patients with positive dipstick tests were referred back to their GP with a view to repeating the appointment in 6 weeks. The remaining 498 phlebotomy appointments were for 485 men (13 men needed repeat PSA tests due to laboratory errors). Twelve men (2%) were between 45 and 49 years old, 24 men (5%) between 50 and 59, and 449 men (93%) between 60 and 69 years old.

Of the 485 men, 68 were considered to have elevated PSA levels—26 in compliance with NICE age‐related thresholds (Cohort 1) and 42 below the age‐related threshold but with a PSA ≥ 3 ng/mL (Cohort 2) (Table 1 and Figure 2).

**TABLE 1:** Summary of characteristics of referred and diagnosed men from Cohort 1 (elevated age‐related prostate‐specific antigen [PSA]) and Cohort 2 (normal age‐related PSA but ≥3 ng/mL).

Characteristic	Referred		Diagnosed	
	Cohort 1 *N* = 26^a^	Cohort 2 *N* = 42^a^	Cohort 1 *N* = 10^a^	Cohort 2 *N* = 8^a^
Age (years)				
50–54	1 (4%)	0 (0%)		
55–59	2 (8%)	0 (0%)	1 (10%)	0 (0%)
60–64	4 (15%)	7 (17%)	0 (0%)	1 (13%)
65–70	19 (73%)	35 (83%)	9 (90%)	7 (88%)
Ethnicity				
Black	1 (4%)	0 (0%)	1 (10%)	0 (0%)
White	24 (92%)	41 (98%)	9 (90%)	8 (100%)
Unknown	1 (4%)	1 (2%)		
Family history of PCa				
No	20 (77%)	33 (79%)	7 (70%)	8 (100%)
Yes	5 (19%)	7 (17%)	2 (20%)	0 (0%)
Unknown	1 (4%)	2 (4.8%)	1 (10%)	0 (0%)
Presenting PSA (ng/mL)	6.05 (5.13, 7.73)	3.40 (3.13, 4.00)	6.80 (6.05, 8.75)	3.65 (3.50, 4.03)
PSA density	0.13 (0.08, 0.17)	0.08 (0.06, 0.11)	0.19 (0.16, 0.23)	0.11 (0.08, 0.13)
Unknown		14^b^		
PI‐RADS				
2	2 (8%)	5 (18%)		
3	16 (62%)	17 (61%)	2 (20%)	3 (43%)
4	5 (19%)	4 (14%)	5 (50%)	2 (29%)
5	3 (12%)	2 (7.1%)	3 (30%)	2 (29%)
Unknown		14^b^		1^b^
ISUP grade group				
1			0 (0%)	1 (13%)
2			6 (60%)	6 (75%)
3			1 (10%)	0 (0%)
4			1 (10%)	0 (0%)
5			2 (20%)	1 (13%)
pT stage				
T2			10 (100%)	8 (100%)

Abbreviations: ISUP, International Society of Urological Pathology; PCa, prostate cancer; PI‐RADS, Prostate Imaging–Reporting and Data System; PT, pathological T stage.

^a^ *n* (%); median (interquartile range [IQR]).

^b^ Fourteen cases referred from Cohort 2 lacked MRI data (eight declined and discharged to GP, four postponed pending PSA review, two lost to follow‐up), one case diagnosed from Cohort 2 lacked PI‐RADS (MRI study limited by bilateral hip prosthesis).

### 3.1 Cohort 1—NICE age‐related PSA threshold

Twenty‐six (5.4%) men had an abnormal age‐related PSA as defined by NICE guidelines and were referred for further assessment under the USC pathway. The mp‐MRI showed PI‐RADS score of 3 in 16 men, and PI‐RADS 4 or 5 in 8 men. Following clinical review, transperineal biopsies were performed in two men with PI‐RADS 3 and in all eight men with PI‐RADS 4 or 5. Of the 14 remaining patients with PI‐RADS 3, one man declined biopsy, two were lost to follow‐up, six were discharged from the Royal Surrey, four were scheduled for a future outpatient appointment and one man remained on PSA surveillance. All 10 biopsies showed clinically significant cancer (six with Gleason 3 + 4, one with Gleason 4 + 3, one with Gleason 3 + 5, two with Gleason 4 + 5). The cancer detection rate for this cohort was 2.1%.

### 3.2 Cohort 2—below age‐related threshold but with PSA at 3 ng/mL or above

In view of the difference between recommended normal PSA levels as advocated by NICE and the NHSE recommendation of PSA ≥ 3 ng/mL for men aged 50–69,^16^ 47 men with a PSA of 3 ng/mL or more but below the NICE age‐related range were separately contacted and offered either an onward USC referral or a repeat PSA check after 6 months.

Sixteen men requested USC referral. After mp‐MRI and clinical review, seven men underwent transperineal biopsies of whom two harboured clinically significant cancer (Gleason 3 + 4 and 4 + 5), one had non‐significant cancer (Gleason 3 + 3).

Twenty‐six men opted for a 6 month repeat PSA test, of these, five chose to be followed up with their GP and were discharged from the pilot study. Of the 21 remaining men in the cohort, five men were diagnosed with clinically significant cancer after the second PSA, clinical review, mp‐MRI and biopsies. All five were Gleason 3 + 4. Thus, eight additional cases were diagnosed using the ≥3 ng/mL cut point, of which one was clinically insignificant. The cancer detection rate for this cohort was 1.6%, and for clinically significant cancer 1.4%.

The overall detection rate in the pilot study was 3.7% for prostate cancer and 3.5% for clinically significant prostate cancer.

## 4 DISCUSSION

Since the early 1990s, the recommendations on the use of PSA to screen for prostate cancer have undergone numerous iterations as evidence from randomised clinical trials continues to mature and diagnostic imaging techniques evolve. The ERSPC followed 182 000 eligible men aged 50 to 74 years identified from population registries randomly assigned to screening every 4 years or no intervention as control. Measurement of PSA in serum, with a cut off of 3.0 ng/mL or more, was the indication for biopsy. Reduction in prostate cancer‐specific mortality was 20% at 16 years of follow‐up (rate ratio [RR] 0.80, 95% confidence interval [CI] 0.72–0.89).^9^

The Göteborg Randomized Population‐Based Prostate Cancer Screening Trial that started earlier then subsequently became incorporated into ERSPC followed 20 000 men aged 50 to 64 with PSA screening every 2 years, cut off >3 ng/mL, and reported 35% reduction in prostate cancer‐specific mortality at 18 years of follow‐up.^10^

Klotz et al. showed that MRI followed by selected targeted biopsy is noninferior to initial systematic biopsy in men at risk for prostate cancer in detecting GG2 or greater cancers.^17^ The use of risk assessment with MRI prior to biopsy, with or without targeted biopsy, was superior to standard transrectal ultrasonography‐guided biopsy.^18^ All in all, the evidence indicates that MRI before biopsy can allow one third of men to avoid an immediate biopsy and reduce overdiagnosis, with 40% fewer clinically unimportant cancers and approximately 15% more clinically important cancers detected.^19^

In view of this new data, the European Union (EU) endorsed the creation of a targeted prostate cancer screening service amongst its member states^20^ and funds the Prostate Cancer Awareness and Initiative for Screening in the European Union (PRAISE‐U) consortium^21^ for which this work will be contributing. The UK Committee on Screening is currently reassessing the situation, and the national charity, Prostate Cancer UK, is supportive of a targeted detection programme.^22^

Various strategies have been reported for case‐finding men at risk for prostate cancer. In this present pilot study, scrutiny of GP records was used to invite 1549 men via SMS text messages. Four hundred eighty‐five men (31%) participated of whom 70 (14%) showed elevated PSA levels and 17 (3.5%) found to have significant (GG ≥ 2) prostate cancer. One man (0.2%) had GG1 cancer.

Similar detection rates were observed in a population‐based screening trial pilot study in Finland.^23^ A group of 399 men randomly sampled from the Finnish population registry were invited to participate by a letter providing written information about prostate cancer, screening and study procedures. All men were asked to sign an informed consent form and fill in a questionnaire (on paper or on the web) about general health, prostate cancer family history, previous PSA and previous prostate biopsies. One hundred and fifty‐eight men (40%) participated and 27 (17%) had PSA levels ≥3 ng/mL. Ten men had a suspicious MRI finding (PI‐RADS ≥3) and 5 men (3%) were diagnosed with clinically significant prostate cancer (GG ≥2) at fusion biopsy. GG1 was diagnosed in 2 men.

Another recent pilot study on a population‐based prostate cancer testing programme in Sweden invited 999 randomly selected men aged 50, 56 or 62 years of whom 418 (42%) men opted for a PSA test.^24^ Participation increased with age, elevated PSA levels (≥3 ng/mL) were observed in 35 men (8%), biopsies were performed in 16 men with PI‐RADS ≥3 and prostate cancer was diagnosed in 10 men—GG ≥ 2 in 7 (1.7%) and GG1 in 3 (0.7%). This detection rate of clinically significant cancer was similar to the 1% reported by the large Swedish Göteborg‐2 trial that screened men with PSA and MRI followed by systematic biopsies and/or targeted biopsies of suspicious lesions shown on MRI.^25^

Moore et al. in the United Kingdom recently took a different approach to case finding by means of mp‐MRI screening of existing patient databases at eight London GP surgeries.^26^ Two thousand and ninety‐six men aged 50 to 75, without a prior prostate cancer diagnosis, were identified and randomly selected for an invitation letter. Of these, 457 men (22%) responded, and 303 men attended for screening MRI. The number of men screened was limited by the availability of MRI slots, so not all eligible responders were able to participate. Forty‐eight men (16%) had a positive MRI, 25 (8.2%) had clinically significant (any Gleason pattern ≥4) cancer and 2 men (0.7%) had clinically insignificant cancer. Fifteen of 25 (60%) men with an abnormal MRI and clinically significant prostate cancer had a PSA < 3 ng/mL.

Measurement of PSA levels remains the first step for detection of prostate cancer. Despite its limitations, success in reducing death from prostate cancer by 20% to 35% (ERSPC^9^ and Goteborg^10^ trials) has led to re‐instatement of PSA screening for prostate cancer amongst EU member states in September 2022. The advent of an MRI national screening programme in the United Kingdom is unlikely to be available in the short to medium term, and its economic viability remains to be determined. Questions also remain as to whether diagnosing men with prostate cancer whilst their PSA is still normal translates to a benefit in cancer survival to offset the side effects of radical treatments. Targeting men with a PSA test followed by mp‐MRI is a sensible first step in detecting prostate cancer at an early stage. At present, this alone would be a major step in the United Kingdom. The ERSCP took over 15 years prior to data becoming mature enough for meaningful analysis. The United Kingdom should adopt the same position as the EU^27^ and create a targeted PSA then MRI detection service possibly with a view in the longer term to screen with MRI if it proves to be both worthwhile and cost‐effective.

General practitioner databases provide a novel way to identify patients at risk, and mobile phone texting is practical in the UK real‐world setting, making this approach a targeted, GP‐record driven process without burdening primary care. There are 111.8 million mobile subscriptions in the United Kingdom, 87 million active mobile devices, and 98% of the adult population have a mobile phone making 1.37 active connections per capita.^28^ The www.talkprostate.co.uk website further raises awareness and provides relevant information and counselling, empowering men to make informed decisions on whether they wish to proceed with the PSA test. Recent evidence indicates that prostate cancer screening does not have an impact on psychosocial health or health‐related quality of life,^29^ hence concerns on whether a targeted approach raises anxiety in invited men seem unwarranted.

This pilot study is by definition a small study, and this is now being rolled out across the cancer alliance to 22 000 men. Our preliminary study was undertaken in a relatively affluent area where engagement may be expected to be higher.^30^ Thirty five percent of men sent an invitation enrolled and had a PSA test in this study compared with the 83% who accepted at least one opportunity to be screened in the ERSPC trial.^9^ The proportion of black men who consented for a PSA test (2.3%) was similar to recent estimates for England of 2.4% black in South East England and 1.7% across the SSCA regions, and our pilot did not capture men with Mixed or Asian ethnic backgrounds that stood at 2.8% for South East England and 3% and 5.8% for the SSCA regions.^31^ Interestingly, race has recently been shown not to be a determinant of PCa‐specific mortality when adjustment for the risk of other‐cause mortality is made.^32^ A more accurate assessment of variation in regional take up rates will be obtained as the programme matures.

In conclusion, our novel Targeted Prostate Cancer Health Check programme identifies men at risk without burdening primary care. This pilot study raised awareness on prostate cancer risk in these men, half of whom received further education upon accessing the programme registration website. One third (30%) of invited men enrolled, and 3.5% were found to have clinically significant prostate cancer. The programme is currently being rolled out across the SSCA.

## AUTHOR CONTRIBUTIONS

Stephen Langley author, study creator and designer, Jeremy Goad clinician and study manager; Stephanie Bell study designer and manager; Lee Foster study and data manager; Catherine Hodges medical adviser and manager; Marc Laniado medical adviser; Michele Pietrasik clinician and data collector and manager; Alison Rooke clinician and data collector and manager; Edward Bosonnet study designer and data manager; Simon Bott clinical adviser and author.

## CONFLICT OF INTEREST STATEMENT

Stephen Langley reports personal fees, non‐financial support and other from BXTAccelyon Limited, outside the submitted work. Jeremy Goad, Stephanie Bell, Lee Foster, Catherine Hodges, Marc Laniado, Michele Pietrasik, Alison Rooke, Matthew Knight, Edward Bosonnet and Simon Bott have nothing to disclose.

## References

[1] Surrey and Sussex Cancer Alliance . Accessed 29 September 2023. https://surreyandsussexcanceralliance.nhs.uk/

[2] Medefer . Accessed 29 Sept 2023. https://medefer.com.

[3] National Institute for Health and Care Excellence . NICE guideline [NG12] Suspected Cancer: recognition and referral. Accessed 29 Sept 2023. https://www.nice.org.uk/guidance/ng12/chapter/Recommendations-organised-by-site-of-cancer#urological-cancers 32212590

[4] The NHS England Clinical Expert Group for prostate cancer: Guidance to support the optimal pathway for prostate cancer, January 2019. Accessed 29 Sept 2023. https://prostatecanceruk.org/media/dadbsf0c/guidance-to-support-the-optimal-timed-pathway-for-prostate-cancer.pdf

[5] https://www.gov.uk/government/publications/prostate-specific-antigen-testing-explanation-and-implementation/advising-well-men-about-the-psa-test-for-prostate-cancer-information-for-gps#psa-test. Accessed Sept 2023.

[6] Office for Health Improvement & Disparities, Guidance, Advisig men without symptoms of prostate disease who ask about the PSA test, Updated 13 May 2022. Accessed 29 Sept 2023. https://www.gov.uk/government/publications/prostate-specific-antigen-testing-explanation-and-implementation/advising-well-men-about-the-psa-test-for-prostate-cancer-information-for-gps#psa-test

[7] Public Health England, Guidance, Prostate cancer risk management programme: overview, updated 29 March 2016. Accessed 29 Sept 2023. https://www.gov.uk/guidance/prostate-cancer-risk-management-programme-overview

[8] Ahmed HU , el‐Shater Bosaily A , Brown LC , Gabe R , Kaplan R , Parmar MK , et al. Diagnostic accuracy of multi‐parametric MRI and TRUS biopsy in prostate cancer (PROMIS): a paired validating confirmatory study. Lancet. 2017;389(10071):815–822. 10.1016/S0140-6736(16)32401-1 28110982

[9] Hugosson J , Roobol MJ , Månsson M , Tammela TLJ , Zappa M , Nelen V , et al. A 16‐yr follow‐up of the European Randomized study of Screening for Prostate Cancer. Eur Urol. 2019;76(1):43–51. 10.1016/j.eururo.2019.02.009 30824296 PMC7513694

[10] Frånlund M , Månsson M , Godtman RA , Aus G , Holmberg E , Kollberg KS , et al. Results from 22 years of followup in the Goteborg Randomized Population‐Based Prostate Cancer Screening Trial. J Urol. 2022;208(2):292–300. 10.1097/JU.0000000000002696 35422134 PMC9275849

[11] Pradere B , Veeratterapillay R , Dimitropoulos K , Yuan Y , Omar MI , MacLennan S , et al. Nonantibiotic strategies for the prevention of infectious complications following prostate biopsy: a systematic review and meta‐analysis. J Urol. 2021;205(3):653–663. 10.1097/JU.0000000000001399 33026903

[12] Tamhankar AS , el‐Taji O , Vasdev N , Foley C , Popert R , Adshead J . The clinical and financial implications of a decade of prostate biopsies in the NHS: analysis of Hospital Episode Statistics data 2008‐2019. BJU Int. 2020;126(1):133–141. 10.1111/bju.15062 32232966

[13] https://www.england.nhs.uk/wp-content/uploads/2018/04/B1348_Prostate-cancer-timed-diagnostic-pathway.pdf. Accessed Sept 2023.

[14] National Health Service . Faster diagnostic pathways, Implementing a timed prostate cancer diagnostic pathway, Guidance for local health and care systems, 21 October 2022. Accessed 29 Sept 2023. https://www.england.nhs.uk/wp-content/uploads/2018/04/B1348_Prostate-cancer-timed-diagnostic-pathway.pdf

[15] Humphrey PA , Moch H , Cubilla AL , Ulbright TM , Reuter VE . The 2016 WHO classification of tumours of the urinary system and male genital organs‐part B: prostate and bladder tumours. Eur Urol. 2016;70(1):106–119. 10.1016/j.eururo.2016.02.028 26996659

[16] https://www.nhs.uk/conditions/prostate-cancer/should-i-have-psa-test/. Accessed Sept 2023.

[17] National Health Service . Should I have a PSA test? Prostate cancer, last reviewed 18 October 2021. Accessed 29 Sept 2023. https://www.nhs.uk/conditions/prostate-cancer/should-i-have-psa-test/

[18] Kasivisvanathan V , Rannikko AS , Borghi M , Panebianco V , Mynderse LA , Vaarala MH , et al. MRI‐targeted or standard biopsy for prostate‐cancer diagnosis. N Engl J Med. 2018;378(19):1767–1777. 10.1056/NEJMoa1801993 29552975 PMC9084630

[19] Drost FH , Osses DF , Nieboer D , Steyerberg EW , Bangma CH , Roobol MJ , et al. Prostate MRI, with or without MRI‐targeted biopsy, and systematic biopsy for detecting prostate cancer. Cochrane Database Syst Rev. 2019;4(4):CD012663. 10.1002/14651858.CD012663.pub2 31022301 PMC6483565

[20] https://health.ec.europa.eu/system/files/2022-09/com_2022-474_act_en.pdf. Accessed Sept 2023.

[21] European Commission . Proposal for a COUNCIL RECOMMENDATION on strengthening prevention through early detection: A new EU approach on cancer screening, replacing Council Recommnedation 2003/878/EC, 20 Sept 2022. Accessed 29 Sept 2023. https://health.ec.europa.eu/system/files/2022-09/com_2022-474_act_en.pdf

[22] https://prostatecanceruk.org/psa-position. Accessed Sept 2023.

[23] Prostate Cancer UK, PSA position. Accessed 29 Sept 2023. https://prostatecanceruk.org/psa-position

[24] Alterbeck M , Thimansson E , Bengtsson J , Baubeta E , Zackrisson S , Bolejko A , et al. A pilot study of an organised population‐based testing programme for prostate cancer. BJU Int. 2023;133(1):87–95. 10.1111/bju.16143 37523331 PMC10787355

[25] Hugosson J , Månsson M , Wallström J , Axcrona U , Carlsson SV , Egevad L , et al. Prostate cancer screening with PSA and MRI followed by targeted biopsy only. N Engl J Med. 2022;387(23):2126–2137. 10.1056/NEJMoa2209454 36477032 PMC9870590

[26] Moore CM , Frangou E , McCartan N , Santaolalla A , Kopcke D , Brembilla G , et al. Prevalence of MRI lesions in men responding to a GP‐led invitation for a prostate health check: a prospective cohort study. BMJ Oncology. 2023;2(1):e000057. 10.1136/bmjonc-2023-000057 PMC11315271 39886504

[27] van Poppel H , Hogenhout R , Albers P , van den Bergh R , Barentsz JO , Roobol MJ . A European model for an organised risk‐stratified early detection programme for prostate cancer. Eur Urol Oncol. 2021;4(5):731–739. 10.1016/j.euo.2021.06.006 34364829

[28] https://www.ofcom.org.uk/__data/assets/pdf_file/0034/264778/Communications-Market-Report-2023.pdf. Accessed Sept 2023.

[29] Ofcom, Communications Market Report 2023. Accessed 29 Sept 2023. https://www.ofcom.org.uk/__data/assets/pdf_file/0034/264778/Communications-Market-Report-2023.pdf

[30] Waterhouse JV , Welch CA , Battisti NML , Sweeting MJ , Paley L , Lambert PC , et al. Geographical variation in underlying social deprivation, cardiovascular and other comorbidities in patients with potentially curable cancers in England: results from a national registry dataset analysis. Clin Oncol (R Coll Radiol). 2023;35(12):e708–e719. 10.1016/j.clon.2023.08.009 37741712

[31] https://www.nomisweb.co.uk/sources/census_2021_bulk. Accessed May 2024.

[32] Office for National Statistics . nomis official census and labour market statistic, Census 2021 Bulk Data Download. Accessed 24 May 13 2024. https://www.nomisweb.co.uk/sources/census_2021_bulk

